# Solitary Tracheobronchial Papilloma: Cytomorphology and ancillary studies with histologic correlation

**DOI:** 10.4103/1742-6413.77286

**Published:** 2011-03-03

**Authors:** Tee U. Lang, Walid E. Khalbuss, Sara E. Monaco, Liron Pantanowitz

**Affiliations:** Department of Pathology, University of Pittsburgh Medical Center, Pittsburgh, PA, USA

**Keywords:** Cytology, dysplasia, human papilloma virus (HPV), lung, papilloma, solitary tracheobronchial papilloma

## Abstract

Solitary tracheobronchial papilloma (STBP) is a rare benign tumor that primarily involves the tracheobronchial tree. Human papilloma virus (HPV) infection is associated with dysplasia and a high risk of carcinoma in these lesions. The cytomorphology of STBP is not well established in the literature. Our aim is to characterize the cytomorphologic features of STBP, with histologic correlation in a series of 6 patients — 4 males and 2 females — with a mean age of 67 years (range, 53-88 years). There were 5 biopsy-proven squamous papillomas and 1 glandular papilloma. On surgical biopsy, squamous papillomas exhibited cytological atypia (4 graded mild and 1 graded moderate with focal severe dysplasia), surface erosion, and inflammation. Cytology specimens available for review included a combination of 4 fine-needle aspirations (FNAs), 2 bronchoalveolar lavages and 2 (of 3) bronchial brushings. Cytologic findings associated with squamous papillomas included atypical squamous cells and rare squamous cell resembling koilocyte in 1 bronchial brushing. Sheets of squamous cells were identified in another specimen. Several cases had a prominent background of acute inflammation, and candida was present in 1 specimen. HPV *in-situ* hybridization was positive in 1 case and negative in 2 cases. A p16 immunocytochemical stain performed on 1 cell block was negative. In conclusion, although STBP is a rare neoplasm, these cases may be encountered in respiratory cytology samples. FNA of papillomas yields fewer lesional cells compared to exfoliative samples. These lesions may be mistaken in cytology specimens for squamous cell carcinoma, squamous-lined cavitary lesions, an infectious (fungal) process, reactive squamous metaplasia, or oral contamination.

## INTRODUCTION

Pulmonary papillomas are a group of rare lung neoplasms that can be divided into 3 types of lesions.[1] The first group consists of multiple papillomatoses, which generally affects the larynx, predominantly in children. Juvenile laryngotracheal papillomatosis rarely involves the lower respiratory tree. These papillomas are related to human papilloma virus (HPV) infection (HPV types 6 and 11 in most cases), recur frequently and have a tendency to regress spontaneously after puberty. The second group of lesions includes inflammatory endobronchial polyps, arising in the mucosa of patients with chronic respiratory infection. These inflammatory polyps are covered by respiratory ciliated epithelium, may have focal squamous metaplasia, and contain an edematous fibrous tissue core with granulation-like tissue and inflammatory cells. The third group, the rarest of the three, comprises the solitary tracheobronchial papillomas (STBP), which represent less than 0.5% of lun g tumors.[2]

STBP usually presents as an endobronchial mass in the segmental bronchi. STBPs can be classified histologically into squamous cell, glandular and mixed (squamous and glandular) type papillomas.[2–5] Squamous papilloma is usually seen in middle-aged male smokers. In contrast, glandular and mixed type papillomas are mostly encountered in older men, and are less commonly associated with tobacco use. Squamous cell papilloma can be exophytic; or, less frequently, inverted. Microscopically they resemble viral papillomas seen in the genito-perineal region. Glandular papillomas (also called columnar cell papillomas) can be lined by ciliated and non-ciliated columnar cells, including occasional goblet cells.[3] Mixed papillomas were previously called transitional papillomas. Patients present either with obstructive symptoms (e.g., wheezing, pneumonia), hemoptysis or are asymptomatic with an incidental finding (e.g., endobronchial protuberance, nodular airway thickening) on radiological imaging. The pathogenesis of squamous cell papilloma, particularly its neoplastic/ tumoral progression to carcinoma, is related to infection with HPV.[2] Although STBPs are noninvasive neoplasms, they are now considered to represent tumors of low malignant potential because they may have dysplasia, recur in around 20% of cases and potentially develop into squamous cell carcinoma.[2]

The cytomorphology of STBP has previously been described in only rare case reports.[6–8] Herein, we review cases of 6 patients to better characterize the cytologic features of STBP, with histologic correlation.

## MATERIALS AND METHODS

A retrospective search of archival cases in our laboratory information system was performed for all cases diagnosed as STBP in respiratory/ thoracic specimens with available histology and cytology material from January 2008 to August 2010. Pediatric patients and all patients diagnosed with multiple papillomatosis and recurrent papillomatosis were excluded. A total of 6 surgical and 9 cytology cases of 6 patients were identified. Available cytology slides for review included 4 fine-needle aspirations (FNAs), 2 bronchoalveolar lavages (BALs) and 2 (of 3) bronchial brushings. All the FNAs were performed transcutaneously, guided by computer tomography (CT), whereas specimens from BALs and brushings were obtained by bronchoscopy. Only 1 patient had multiple cytology specimen types, including a repeat FNA procedure. Data regarding patient demographics, papilloma anatomical location, cytology findings, histology findings and the results of any ancillary studies are tabulated in Table 1. The details regarding the clinical presentation and imaging findings were unavailable for this study. The data were analyzed using descriptive statistics. Institutional review board approval was obtained for this study.

**Table 1:** Summary of clinical, histopathological and cytological features of STBP

*Case no. (reference)*	*Age (years)*	*Gender*	*Airway location*	*Specimen type*	*Histology features*	*Squamous dysplasia grade*	*Cytology specimen*	*Cytology features*	*HPV result*
1	53	Male	Right lower main stem bronchus	Biopsy	Squamous papilloma	Moderate and focal severe dysplasia	FNA #1	Atypical squamous cells	HPV positive
							FNA #2	Atypical squamous cells	
							BAL	Atypical squamous cells	
							Bronchial brushing	Atypical squamous cells and one squamous cell with koilocytosis	
2	65	Male	Right upper main stem bronchus	Biopsy	Squamous papilloma	Mild dysplasia	Bronchial brushing	No atypical cells. Benign bronchial epithelial cells	Not available
3	85	Male	Left main bronchus	Biopsy	Glandular papilloma	No dysplasia	FNA	No atypical cells. Benign bronchial epithelial cells	Not available
4	56	Male	Right upper main stem bronchus	Biopsy	Squamous papilloma	Mild dysplasia	FNA	No atypical cells. Benign bronchial epithelial cells	Not available
5	88	Female	Left lower main stem bronchus	Biopsy	Squamous papilloma	Mild dysplasia	BAL	Atypical squamous cells, Candida present	HPV negative
6	56	Female	Distal trachea	Biopsy	Squamous papilloma	Mild dysplasia	Bronchial brushing	Atypical squamous cells	HPV negative
7[6]	46	Female	Left lower bronchus	Lobectomy	Squamous papilloma	No dysplasia	Tumor imprint	Benign squamous cells	Not available
8[7]	29	Male	Right lower lobe, located peripheral	Lobectomy	Squamous papilloma with mucous gland adenoma	No dysplasia	FNA	Benign squamous cells	Not available
9[8]	59	Male	Left lower bronchus	Lobectomy	Mixed squamous and glandular papilloma	Mild dysplasia	Bronchial brushing	Atypical squamous cells, ciliated glandular cells	HPV negative
							Tumor imprint	Atypical squamous cells, ciliated glandular cells	

(Cases 1 through 6 are from the current series. Cases 7, 8 and 9 are from previously published case reports)

## RESULTS

### Clinicopathological findings

There were 4 males and 2 females with a mean age of 67 years (range, 53-88 years). Five papillomas were located in the bronchus (3 right main stem, 2 left main stem) and 1 case was found growing in the trachea. Clinical details pertaining to these cases were not available for review.

### Cytomorphology findings

**Figure 1:**
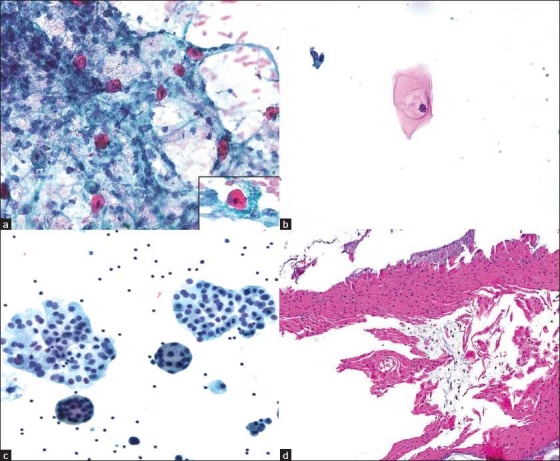
Cytomorphology of solitary tracheobronchial papillomas; a) Atypical squamous cells present in a background of neutrophils (Case 1, ×40 magnification; Pap stain smear), with atypical squamous cell (inset, ×60 magnification); b) Squamous cell resembling a koilocyte identified in a bronchial brushing specimen (Case 1, ×40 magnification; Pap stain, ThinPrep); c) Ball of squamous cells seen on ThinPrep (Case 6, ×40 magnification; Pap stain, ThinPrep); d) Sheet of squamous cells seen in cell block (Case 6, ×10 magnification; H and E stain, cell block)

Cytology slides showed moderate cellularity, except for 2 (50%) out of 4 FNA cases, which were suboptimal. The cytology specimen types and their final diagnoses are summarized in Table 1, and the cytologic features are summarized in Table 2. The most common cytological finding was an acute inflammatory background with abundant neutrophils, in 4 cases [Figure 1a]. A subset of cases also had chronic inflammatory cells, including lymphocytes and macrophages. The FNA performed in the single case with a glandular papilloma was interpreted as nondiagnostic. In cases with a squamous papilloma, there were scattered and loose clusters of keratinizing and non-keratinizing squamous cells. Although most of these cases contained benign squamous cells with dark pyknotic nuclei, admixed atypical squamous cells were also noted. One squamous cell resembling a koilocyte was seen in 1 case [Figure 1b]. Although this cell has a distinct perinuclear halo, marked nuclear atypia was not a feature. In 1 case diagnosed with squamous papilloma, the bronchial brushing showed balls of squamous cells [Figure 1c] and a large sheet of squamous cells in the cell block [Figure 1d]. In 1 BAL specimen, fungal organism morphology compatible with c andida species was identified.

**Table 2:** Summary of cytological features of STBP

*Specimen type*	*Cytological features*	*Positive/ Available slides (%)*
FNA	Cellularity – moderate	2/4 (50)
	Inflammatory cells – neutrophils	2/4 (50)
	Epithelial cell type – squamous cells	2/4 (50)
	Atypia – mild	2/4 (50)
	Koilocytes	0/4
Bronchial brushing	Cellularity – moderate	2/3 (66)
	Inflammatory cells – neutrophils	2/3 (66)
	Epithelial cell type – squamous cells	2/3 (66)
	Atypia – mild	2/3 (66)
	Koilocytes	l/3 (33)
Bronchoalveolar lavage	Cellularity – moderate	2/2 (l00)
	Inflammatory cells – neutrophils	2/2 (l00)
	Epithelial cell type – squamous cells	2/2 (l00)
	Atypia - mild	2/2 (l00)
	Koilocytes	0/2

### Histopathology findings

**Figure 2:**
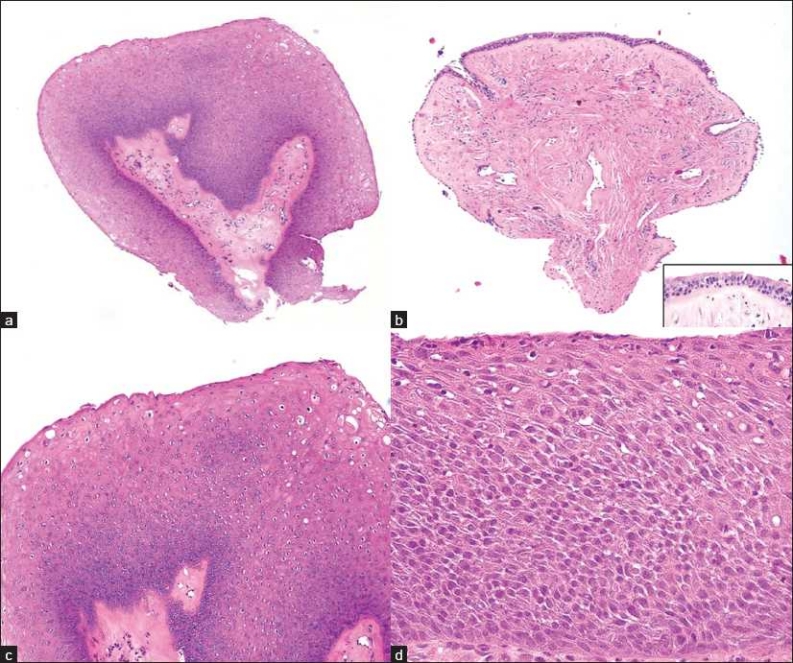
Histopathology of solitary tracheobronchial papillomas; a) Papilloma lined by squamous epithelium (Case 5, ×10 magnification; H and E stain); b) Papilloma lined by glandular epithelium (Case 3, ×10 magnification; H and E stain), with ciliated columnar cells (inset, ×40 magnification); c) Squamous epithelium with low-grade dysplasia (Case 5, ×20 magnification; H and E stain); d) Squamous epithelium with high-grade dysplasia (Case 1, ×40 magnification; H and E stain)

There were 5 cases of squamous papillomas and 1 case of glandular papilloma. Most papillomas had stromal cores. The squamous papilloma was lined by squamous epithelium with acanthosis and keratinization of the surface [Figure 2a], whereas the glandular papilloma was lined by glandular epithelium [Figure 2b] consisting of ciliated columnar cells [Figure 2b inset]. Varying degrees of cytological nuclear atypia were noted in all of the squamous papillomas: 4 had mild squamous dysplasia [Figure 2c], and 1 had moderate squamous dysplasia [Figure 2d]. Nuclear atypia was not present in the glandular papilloma. Surface erosion was identified in 3 cases. Acute and chronic inflammation was noted in 3 and 2 cases, respectively. Ulceration, necrosis or carcinoma was not a feature in any of these cases.

### Ancillary study results

**Figure 3:**
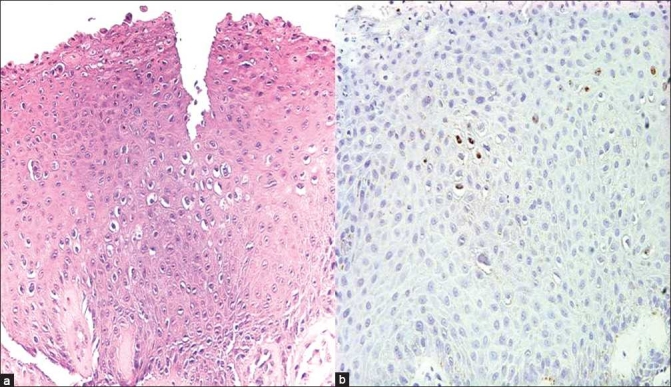
a) Histopathology of squamous epithelium showing koilocytes (Case 1, ×40 magnification; H and E stain); b) HPV *in-situ* hybridization in a squamous papilloma with dysplasia, showing focal nuclear positivity (Case 1, ×40 magnification; p16INK4, MTM lab)

*In-situ* hybridization for HPV DNA (HPV DNA probe, Dako) was performed in 3 of the surgical biopsy specimens. HPV was positive in only 1 of these cases [Figure 3]. An immunohistochemical stain for p16 (p16INK4, MTM lab) performed on 1 case with cell block material was negative.

## DISCUSSION

Given that STBP is a rare neoplasm, it is not surprising that the cytomorphology of these tumors is not well characterized. Although our study contains only a limited number of cases, to the best of our knowledge, this is the largest reported series of cases that describe the cytomorphologic features of STBP. A review of the literature yielded only 2 prior articles describing the cytological features of a squamous papilloma,[6,7] and 1 article on a mixed squamous and glandular papilloma of the lung.[8] In the first case report with a squamous papilloma, the left lower lobectomy in a 46-year-old patient showed a 2-cm ‘wart-like’ excrescence. A tumor imprint of the papilloma was performed, which was composed of squamous cells with abundant cytoplasm but without nuclear atypia or irregularity.[6] The second case report described a 1.5-cm squamous papilloma with a mucous gland adenoma in right lobectomy in a 29-year-old man. The FNA smears showed papillary clusters of benign squamous cells that were interpreted as reactive squamous metaplastic cells.[7] More recently, a case of a mixed squamous and glandular papilloma was reported in a 59-year-old man who presented with persistent cough, bloody sputum and a 2-cm mass in his left lower lobe.[8] A bronchial brush cytology smear in this individual was reported to be of moderate cellularity and consisted of a mixture of squamous and columnar cells. The squamous cells exhibited round nuclei with mild nuclear atypia, cytoplasmic keratinization and dark pyknotic nuclei. The columnar cells showed cilia and lacked atypia. The cytologic features of the squamous cells described in all 3 cases were similar to those seen in our cases.

STBP may be encountered in both FNA material and exfoliative respiratory specimens. In practice, it is challenging to render a definitive diagnosis of STBP on cytology specimens, because these cases lack specific distinguishing cytomorphological features. Since glandular papillomas are lined by ciliated bronchial epithelium, the finding of these normal glandular cells may be interpreted as nondiagnostic in the context of a lung mass, as occurred in 1 of our cases. Of all the cases in this study, the diagnosis of a papilloma was raised only in the bronchial brushing specimen with large fragments of papilloma in the cell block. Most of the remaining specimens with adequate cellularity were called ‘atypical’ due to the finding of atypical squamous cells. This is not surprising, given that all squamous papillomas in our series had varying degrees of dysplasia. Other diagnoses included in the differential of squamous papilloma are squamous cell carcinoma, squamous-lined cavitary lesions, an infectious (fungal) process, reactive squamous metaplasia, as well as squamous cells that are due to contamination from the upper respiratory tract or oral cavity. It is unclear if the presence of candida in our BAL case was related to the papilloma or contamination. Even clinically, it may be difficult to distinguish squamous papilloma from other papillary lesions by bronchoscopy, including squamous cell carcinoma, as both have exophytic growth into the bronchial lumen. There have been documented cases of malignant change arising in STBP, and squamous cell carcinomas were found to be the commonest malignancy.[9,10] In cytology specimens, squamous cell carcinoma may be difficult to distinguish from papillomas with dysplasia. The presence of squamous cell carcinoma arising in a papilloma will require histopathological examination.

HPV has been strongly linked to the pathogenesis of STBP. Specifically, HPV types 6 and 11 are most commonly seen in STBP, with benign features and favorable outcome; whereas HPV types 16 and 18, sometimes in combination with HPV 31/33/35, are associated with STBP that have a higher risk of carcinomatous transformation.[11,12] In our study, a rare squamous cell resembling a koilocyte and possibly indicative of HPV infection of a papilloma was identified in a bronchial brushing. Moreover, *in-situ* hybridization for HPV was positive in 1 (33%) of the 3 cases of squamous papilloma. In another series, positive HPV DNA was reported by investigators in 5 (71%) of 7 squamous papillomas[13] ; as well as by other authors, in squamous papillomas with[11,14] and without koilocytosis.[15] The diagnostic utility of HPV studies and surrogate biomarkers like p16 in STBP has not been well documented.

In summary, although rare, STBP may be encountered in respiratory cytology samples. STBP may be difficult to diagnose based on cytomorphology alone, and our experience revealed that exfoliative samples yield more lesional cells compared to FNA. It is unclear whether the difference in cell yield is directly related to the technical approach in sampling the lesion — with a more direct sampling method using BAL or brushing in comparison to targeting a specific area by FNA. However, in the clinical (bronchoscopic) context of a solitary papillary lung lesion, the presence of koilocytes among atypical squamous cells in respiratory cytology samples and positive HPV ancillary studies, the diagnostic possibility of an STBP should be entertained. The finding of squamous cells with parakeratosis, cytologic atypia and HPV-related cytopathic effect in lung cytology due to STBP should not be misinterpreted as squamous cell carcinoma.

## COMPETING INTEREST STATEMENT BY ALL AUTHORS

The authors declare that they have no competing interests.

## AUTHORSHIP STATEMENT BY ALL AUTHORS

All authors of this article declare that we qualify for authorship as defined by ICMJE. All authors are responsible for the conception of this study, participated in its design and coordination, and helped to draft the manuscript. All authors read and approved the final manuscript.

## ETHICS STATEMENT BY ALL AUTHORS

This study was conducted with approval from Institutional Review Board (IRB) (or its equivalent) of all the institutions associated with this study. Authors take responsibility to maintain relevant documentation in this respect.

## EDITORIAL / PEER-REVIEW STATEMENT

To ensure integrity and highest quality of CytoJournal publications, the review process of this manuscript was conducted under a double blind model(authors are blinded for reviewers and reviewers are blinded for authors)through automatic online system.
